# Ether Inhalation in Convulsions

**Published:** 1848-04

**Authors:** Richard C. Wyatt

**Affiliations:** Shelby county, Tennessee


### WESTERN JOURNAL

OF

### MEDICINE AND SURGERY.

APRIL, 1848.

Art. I.—*Ether Inhalation in Convulsions.* By Richard C. Wyatt, M.D., of Shelby county, Tennessee.

The extended use and happy influences of the sulphuric ether by inhalation, seem to be exciting the attention of the medical profession generally. The annexed report of its administration in a case of convulsions, from gastro-enteric irritation, may, I hope, be serviceable in proving its yet more extensive applicability in relieving, or at least mitigating, the anguish of suffering humanity.

In regard to the effects of the ether, in this case, the report shows that it was not entirely successful in preventing the convulsions, but that it greatly mitigated their severity, shortened their duration, and really lessened their number. I will here add that my apparatus was very imperfect, the vapor being so concentrated, on first dropping through the reed as to produce a sense of suffocation, and an inability to inhale it; but in a minute more, becoming so weak from rapid evaporation, as to have but little effect. But imperfect as it was, I am fully satisfied that several paroxysms were prevented, and many others cut short, by its continued administration. And it seems very probable, that with a perfect apparatus and proper attention, they might all have been prevented, after commencing its use. Its influence on the pulse was prompt and decided,—lessening its frequency and increasing its fulness, to a remarkable extent.

Allow me to remark, that the case was not designed to be reported, until after the ether had been used, and as I had preserved only a general outline of its history, progress, and treatment, there is necessarily a want of fulness and precision in its details, which robs it of a portion of its interest.

J. B. G., aged 11 years, of weakly constitution, and rather delicate health, was taken, on the 3d or 4th of December, with sick stomach, vomiting, and constipation of the bowels, attended with slight fever. Having been, some two years before, severely and dangerously salivated, and his system subsequetly proven unusually susceptible to the constitutional influence of mercury, his parents were unwilling to resort to it again, if it could possibly be avoided. They gave him an emetic, with some other simple remedies at their command, but without effecting any improvement in his condition.

7th. I was summoned to visit him, and found him with severe fever; pulse neither quick nor very frequent; tongue red, and somewhat furred; nausea; occasional vomiting, and considerable soreness and tenderness, on pressure, of the epigastric and hypochondriac regions. Bowels constipated. ℞. Blue mass, v. to viii. grs., to be repeated at intervals of two hours. Large pepper poultice to be applied to the abdomen, and during the evening injections to be administered, and repeated at short intervals, till his bowels are moved.

8th. No action on the bowels; vomiting continues, with increased irritability of the stomach; skin generally cool; pulse rather weaker, and perhaps a little increased in frequency; no secretory action manifest. Considerable prostration of strength, and of all the vital energies of the system. Gave him calomel ii.grs., sulph. morph. ¼gr; repeated at two hours' interval, till the third dose was taken. Sinapisms to the epigastrium and extremities; and injections continued, and repeated at short intervals till his bowels are opened.

9th—Morning. Patient grows worse. No action yet on his bowels, and apparently an entire suspension of all the secretions. Tenderness of the abdomen continues; thirst considerable, but almost incessant vomiting. The stomach rejects alike, medicine, aliment, and drinks of every description. Skin still cool; pulse weakened, and prostration extreme. Gave him calomel v.grs., and occasionally a little camphor julep and carb. ammonia, in a mucilage of gum arabic. Applied blisters to the epigastrium, and sinapisms to the extremities, to be frequently renewed and continued through the day.

Evening. Some slight evidence of ptyalism, but no reaction of system. Vomiting continues; skin cool; pulse, at times, imperceptible at the wrist. Complains of a little pain in the head, elicited from inquiry, on observing some contraction of brow, and slight dilatation of the pupil of the eyes. Gave him brandy toddy as often as his stomach would receive it, and continued the persevering application of mustard plasters.

11 o'clock, P. M. Injections operated on his bowels, bringing away a considerable discharge of fecal matter, after which he rested pretty quietly and slept.

10th—Morning. Patient apparently much better. Vomiting ceased; circulation nearly restored; desires food; soreness of mouth increased, with several small mercurial blisters on the tongue and cheeks; flow of saliva sufficiently free.

Evening. Somewhat unnatural expression of countenance; slight dilatation of the pupils of his eyes; occasional symptoms of strangury. ℞ Spirits nitre in demulcent and mucilaginous drinks.

11th. Summoned to see my patient, who is reported much worse, and "about to die." Learned that during the night he had been taken with convulsions, and had several before my arrival. Found him with pulse accelerated, but still weak; skin generally cool; pupils of his eyes largely dilated; indistinctness of vision; mind entirely rational. No action on the bowels since the one of Thursday night. Stomach irritable, and though not vomiting so incessantly as a few days before, yet rejecting every thing in the shape of medicine, even to gum water and rice gruel. He was taken with a convulsion a few minutes after my arrival. It commenced in the right hand and arm, but extended almost instantly to the trunk and head. Spasmodic action, particularly of the muscles of the face and superior extremities, violent and alarming. Duration of the paroxysm, probably five to eight minutes—going off gradually. Arterial action, during its continuance very perceptibly weakened. Enquired, as soon as he could speak, what was the matter with him. The convulsions being caused apparently by gastro-enteric irritation, aided probably, to some extent, by venous congestion of the brain, and no evidence of any inflammatory action in this organ manifest. And again; the condition of the patient requiring a stimulant as well as antispasmodic, it occurred to me that it was a fit case for the trial of sulphuric ether by inhalation. I prepared immediately for its administration; and by the time my apparatus (a reed secured in the inner fold of a thickly folded linen) was ready, had an opportunity of testing its virtues. Another convulsion threatening more violence than the last, coming on, the application was made—the linen being kept supplied through the reed. In less than a minute, I think, probably less than a dozen inspirations, the convulsive muscular action ceased, almost instantaneously, leaving the patient in quiet repose. Several persons present, amongst them a medical friend, were all agreebly surprised at its prompt and happy effect. Throughout the evening there was a frequent recurrence of the convulsions, with sometimes only a few minutes' interval between the attacks; but as often would the ether, when properly administered, cut short their duration, and frequently, when used in time, prevent the paroxysm altogether. Indeed so evident was its salutary influence that the patient himself, after a few trials, would call for it eagerly and hastily on perceiving the approach of the fit. All secretory action having been again suspended, I had given him, about noon, some eight or ten grains pil. hydrarg.—applied blisters to the back of his neck, sinapisms to the extremities, and directed the frequent repetition of injections to open his bowels.

11 o'clock, P.M. No action of the bowels Blisters had drawn well, but again produed strangury. Pulse still weaker; and convulsions continue to recur. The ether giving out, he had some ten or twelve in an hour, very violent, and succeeding each other so rapidly as scarcely to give one time to go off before another would come on. A new supply of ether was received as the paroxysms began to lessen in violence and freqency; used it with the same success as before, but patient slightly delirious for some two or three hours, after which he rested, and occasionally slept.

12th—Morning. Patient free from delirium, but convulsions continue to recur—attacking now the left hand and arm, but extending directly to the trunk and head, as before. Arterial action quite weak, and pupils of his eyes largely dilated. No action yet on the bowels. Gave him three drops of croton oil in sweet oil and mucilage of gum arabic—rejected it by vomiting. Again five drops, on loaf sugar, but this soon followed the other. Continued sinapisms to the extremities; warm cataplasms to the abdomen, and the ether vapor for the convulsions.

In the evening, a violent paroxysm of strangury and restlessness was speedily relieved, by putting him fully under the influence of the ether; after which, he rested quietly some two hours.

At night, a copious discharge of urine. Rested better this, than during the preceding night.

13th. Patient's bowels freely opened by injections. Less frequency, and more fulness of pulse. Convulsions ceased, but frequent spasmodic twitches of the tendons of right hand and arm. Pupils of eyes a good deal dilated yet; says he feels much better; and gives some attention (which he did not yesterday) to surrounding objects. Desires food; but profiting by past experience, gave him less latitude, confining him rigidly to rice gruel as diet and drink.

14th. Found him still improving; rested well through the night; bowels opened by injections; still desiring food. Pulse yet rather weak, and pupils of his eyes continue dilated. Continue the rice gruel, with occasionally a little brandy and water.

15th. Patient improving. Appetite good; pupils of his eyes less dilated; pulse fuller, stronger, and less frequent; bowels open. Allowed chicken broth for his diet.

After the date above, my patient continued to convalesce, with some little attention to regimen and the condition of his bowels; yet was it nearly a week before he was able to walk, even about his room, though after this convalescence went on more rapidly, and he was soon able to return to his school.

Shelby county, Tenn., December 29, 1847.

